# What motivates senior clinicians to teach medical students?

**DOI:** 10.1186/1472-6920-5-27

**Published:** 2005-07-18

**Authors:** Jane Dahlstrom, Anna Dorai-Raj, Darryl McGill, Cathy Owen, Kathleen Tymms, D Ashley R Watson

**Affiliations:** 1 Medical School, Building 42A, ANU, Canberra, ACT 0200, Australia

## Abstract

**Background:**

This study was designed to assess the motivations of senior medical clinicians to teach medical students. This understanding could improve the recruitment and retention of important clinical teachers.

**Methods:**

The study group was 101 senior medical clinicians registered on a teaching list for a medical school teaching hospital (The Canberra Hospital, ACT, Australia). Their motivations to teach medical students were assessed applying Q methodology.

**Results:**

Of the 75 participants, 18 (24%) were female and 57 (76%) were male. The age distribution was as follows: 30–40 years = 16 participants (21.3%), 41–55 years = 46 participants (61.3%) and >55 years = 13 participants (17.3%). Most participants (n = 48, 64%) were staff specialists and 27 (36%) were visiting medical officers. Half of the participants were internists (n = 39, 52%), 12 (16%) were surgeons, and 24 (32%) were other sub-specialists. Of the 26 senior clinicians that did not participate, two were women; 15 were visiting medical officers and 11 were staff specialists; 16 were internists, 9 were surgeons and there was one other sub-specialist. The majority of these non-participating clinicians fell in the 41–55 year age group. The participating clinicians were moderately homogenous in their responses. Factor analysis produced 4 factors: one summarising positive motivations for teaching and three capturing impediments for teaching. The main factors influencing motivation to teach medical students were intrinsic issues such as altruism, intellectual satisfaction, personal skills and truth seeking. The reasons for not teaching included no strong involvement in course design, a heavy clinical load or feeling it was a waste of time.

**Conclusion:**

This study provides some insights into factors that may be utilised in the design of teaching programs that meet teacher motivations and ultimately enhance the effectiveness of the medical teaching workforce.

## Background

Clinical teachers are central to the successful education of medical graduates. They are a precious resource with a range of competing activities like clinical care and research. In order to better recruit and retain clinical teachers, medical schools must be cognisant of the variety of factors that may motivate doctors to teach students. Medical schools have increased expectations of clinical teachers with curriculum development, small group dynamic teaching and assessment responsibilities, yet have little direct line management of clinical teachers. In Australia, universities do not pay their clinical teachers and therefore they do not "own" them. Hospitals pay doctors and implicitly expect them to teach as a service to the profession. There is no clearly stated contractual requirement. Substantial resource is put into training clinical teachers and schools are interested in minimising teacher turnover. Initial motivation and any accompanying rewards are central to remaining motivated to teach. Equally medical schools have an incentive to recruit satisfied and effective teachers in order to improve educational outcomes.

A motive can be defined as an entity that impels one to action of a particular type. In contemporary psychology, motivation encompasses three areas: drives (innate origins of behaviour that impel an individual to action), goals or purpose (a conscious plan for action that entices an individual to action) and reinforcers (entities that increase or decrease the probability of a behaviour being replicated such as pleasure/pain or reward/punishment) [1]. Motivation is a complex concept investigated in a number of ways. Maslow identified five levels of "needs" or drives that motivate behaviour: physiological needs (to satisfy hunger, thirst, shelter), safety needs (for security, order and stability), belonging and love needs (affection and identification), esteem needs (prestige, success and self-respect) and the need for self actualisation [2]. Goals may be achievement oriented (such as meeting a set of learning goals) or prosocial goals (such as peer acceptance and respectability) [3]. Herzberg, (quoted in Ellis) contrasted extrinsic rewards (pay and benefits) and the inherent intrinsic rewards (such as self respect and personal achievement) [4]. These three areas of drives, goals and rewards all overlap and influence behaviour in different ways at different times.

So what is understood of the motivations of clinical teachers? Clinical supervisors rated predetermined possible motivations to explain their volunteering to teach: personal satisfaction was highest, followed by the opportunity to attract students to one's speciality area. Less important was any sense of prestige or improved standing amongst peers. Despite focusing on an intrinsic motivator, this group still identified a need to be acknowledged by the medical school. Faculty appointments, discounted continuing education, access to computerised information and libraries along with better education as clinical teachers were all valued as suitable rewards. Supervisors in open responses did not suggest monetary compensation [5]. Work focusing on medical teachers' reluctance to teach noted ten impediments including lack of reward, perverse incentives of academic promotion by research with little value on teaching, lack of teaching skill, competitive agendas of clinical service and research, obtuse curriculum redesign and administrative blocks like high student teacher ratios [6]. Broader study on motivation for academic career progression amongst academic physicians found gender and work focus (research vs teaching) influenced motivation. Compared with male physicians, female physicians were more motivated in work by the desire to help others while clinician researchers valued self-expression as a more powerful motivator than did clinician educators [7].

A review of rewards and incentives for non-salaried clinical teachers found that most medical schools offered some incentive such as educational opportunities, academic appointments and special recognition events [8]. Medical faculty (directly paid by a university to teach) valued recognition of outstanding teaching (the Dean's teaching awards) and educational development as a teacher as suitable reward [9].

This study applied Q methodology, an established sorting method, to quantify subjective views on motivation. This method has previously successfully screened aspiring schoolteachers as to why they chose a career in teaching [10] (accessed 16/02/2002) and reviewed career satisfaction amongst nurses [11].

We aimed to investigate what motivates our senior clinicians to teach medical students in order to identify factors that may assist in the design of teaching programs that meet teacher motivations and ultimately enhance the effectiveness of the medical teaching workforce.

## Methods

### Study group

In our context, this work was quality improvement in education and did not require formal ethics approval. The study group was all senior clinicians registered on the medical student teaching staff list at a public, tertiary level regional teaching hospital (The Canberra Hospital, Canberra, Australia). Their appearance on the list implied some interest in teaching. Demographic data collected included gender, age group (30–40, 41–55, >56 years), employment contract (salaried staff specialist or contracting visiting medical officer), and the speciality of the clinician (internist, surgeon, or other sub-specialist – pathologists, radiologists, psychiatrists etc.).

### Q methodology

This method was chosen as the assessment and statistical method for the study as it combines qualitative and quantitative research traditions [12] (accessed 07/06/2005). Q methodology can reveal the subjectivity in a situation and although initially used in personality assessment, has been applied to a range of psychological investigations [13]. The method allows a quantitative evaluation of the opinion of individuals about topics of common concern. This leads to a composite of opinions that may be aggregated into viewpoints [14].

Central to Q methodology is the ranking of single statements on a continuum detailing the degree of agreement or disagreement with the statement. Unlike single dimension questionnaires, the use of a quasinormal distribution forces participants to rank statements relative to the other statements about the question of concern. The ranking of individual opinions about the statements facilitates the formation of individual viewpoints about the overall subjective question to which the sample statements referred. The viewpoints are then compared in a correlation matrix to identify similarities between individual viewpoints.

### Choosing the sample population (P-set)

Individuals are chosen to participate in a study based on their relevance to the goals of the study, as opposed to being selected for their representativeness of a larger population. This collection of individuals is referred to as a "person-set", or P-set [15]. The P-set for this study was drawn from senior clinicians on the teaching list of the Canberra Clinical School at The Canberra Hospital (n = 101). Of these, 26 were on leave or not contactable during the time agreed for data collection. A limited time frame for the data collection was required to limit staff discussion of the study between participants before providing their opinion. All 75 contactable staff agreed to participate. Apart from instructing the participant on the study objectives, the method used and how to complete the questionnaire, there was no other discussion between the participant and the researcher.

### Creating the Q sample

This is the creation of statements used to examine the topic of investigation. These need to be drawn from people with expertise in the issue under study. They may be developed through focus group discussions or brainstorming [11]. The clinicians conducting the study (which represented the population of interest i.e. senior teaching clinicians) contributed statements representing reasons clinicians may teach, or not teach, medical students. All members were experienced clinical teachers undertaking further studies in medical education. From the study group deliberations and through examination of the relevant literature, a representative set of 69 statements (the Q sample) was created (Table 1). Approximately equal numbers of positive and negative statements were created. The statements were consecutively numbered and printed onto labels. The labels were then put onto note cards with one statement per card.

**Table 1:** Statements included in the Q sort

**Statement**
1. I enjoy spending time with students in small groups
2. I don't enjoy lecturing to large groups of students
3. I like the challenge of teaching students as effectively as possible
4. I am bored by teaching
5. I don't feel any sense of duty to teach
6. I teach because it sets a good example to my students to become teachers
7. I teach because I have been inspired to teach by my mentors
8. I teach because I am good at it relative to other academic skills
9. I teach because it is a requirement of my employment contract
10. I teach because I believe it is an appropriate service to my profession
11. Teaching doesn't do anything to enhance my clinical knowledge and/or skills
12. I teach because I enjoy the sense of performing in front of an audience
13. I don't get any financial reward from teaching
14. I teach because I want to help my students become good doctors
15. I don't teach because I am not the one most familiar with a given topic
16. I don't teach because my institution provides poor facilities for teaching
17. I don't teach because I have insufficient time available to teach
18. I teach because there are opportunities for 'virtual' and/or 'online' and/or remote teaching
19. I don't teach as my speciality is too 'cutting edge' to be relevant to students
20. I don't teach because there are no clearly stated learning goals in the course
21. I don't teach because there is no strong involvement of teaching staff in the design of the course
22. I don't teach because there is no recognition for what I do
23. Opportunities for academic promotion have nothing to do with my motivation to teach
24. I teach because the course allows a deep approach to learning by the students
25. I don't teach as students make me feel inadequate
26. I don't teach because opportunities are not available for me to improve my teaching skills
27. I don't teach because I receive inadequate feedback from students on my performance
28. I teach because I believe I communicate well with people
29. I don't teach because I believe the institution devalues teaching and learning
30. I don't teach because the setting in which I am expected to teach is inappropriate
31. I teach because I feel part of the continuum of learning of my students' experience
32. I teach because I feel responsible for the student learning outcomes of my efforts
33. I teach because it gives me a sense of power
34. I teach to improve my communication skills
35. I don't teach as I am not a useful role model
36. My clinical load deters me from teaching
37. My clinical load deters me from putting any time into preparation for teaching
38. I don't teach because I am not concerned about the success of the clinical and/or medical school
39. The teaching I had as a medical student has inspired me to want to teach
40. I teach as a means of reviewing a topic area unfamiliar to me
41. I teach to be challenged in my established views
42. I don't teach because I find it unenjoyable
43. It teach because of the prestige it gives me with my peers
44. I teach because my patients expect it of me
45. I don't teach because interacting with students is boring
46. I teach because of the intellectual stimulation
47. I teach because my colleagues expect me to do so
48. I teach because I was asked to do so by the Clinical and/or Medical School
49. I don't teach because it fails to keep me up to date
50. I teach students because interaction with them makes me think more critically
51. I teach students to ensure they receive a balanced clinical education.
52. I teach because I can enhance my knowledge and understanding of junior doctors
53. I teach because the interaction with students provides an opportunity for my opinions to be heard
54. I teach to ensure the students appreciate my specialty in a favourable way
55. I teach because it allows me to interact with students and show an appreciation of their position
56. I don't teach just because it is expected of me
57. I teach students to show them the correct way of clinical practice in my specialty
58. I teach to ensure any false understanding of my specialty is not perpetuated
59. I teach because I can demonstrate a healthy lifestyle to my students
60. I don't teach just because of the academic position I hold
61. I teach because I can challenge students to be more critical in their thinking.
62. I don't teach because one can't influence the behaviour of students for the better
63. I don't teach because teachers don't contribute to the formation of future doctors
64. I don't teach as I don't approve of new teaching techniques
65. I don't teach as students today lack respect
66. I don't teach as it is a waste of time
67. I teach to engage with younger people
68. I teach as it enhances my status in my profession
69. I don't teach as I feel my knowledge is out of date

### Creating the Q sort and data collection

**Figure 1:**
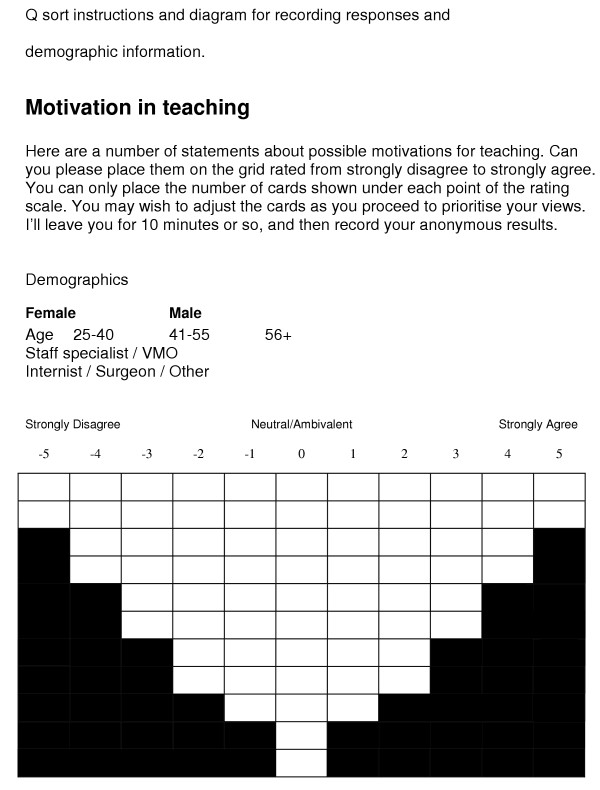


After formation of the Q sample, a quasi-normal distribution containing as many cells as there were Q sample statements was created [11]. A table (11 columns by 11 rows) was made and then reduced to a series of cells in a symmetrical forced normal distribution (Figure 1). This distribution was referred to as a Q sort and was the data collection instrument. A cover sheet also contained an outline of the purpose of the study, instructions to participants and recorded demographic data. An enlarged version of the Q sort was created to fit the note cards to facilitate the physical sorting of statements (Q sort diagram).

Each member of the group approached an allocated subset of the senior medical staff to request their participation. After a brief discussion of the methodology each consenting participant was then asked to undertake the study at a convenient time and place. Each participant was given the stack of 69 note cards containing the questions and instructed to sort all cards according to their level of agreement or disagreement onto the large Q sort diagram. The 69 note cards with each statement were placed by the clinician on the ranking space perceived as most appropriate for that individual statement in comparison to the other statements. This manual form of positioning of the note card with a statement on it allowed the ranking of a statement to be altered easily when comparing it to each new statement assessed by the participant. The final positioning of all statements therefore provided a ranking of the statements relative to each other in importance as determined by the individual clinician.

Demographic information was collected on each participant. The Q sample statements and data from the participants were entered into the Q methodology freeware program downloaded from the Q Method Page [16] (accessed 03/06/2002).

### Data analysis

Factor analysis using the Centroid approach with varimax rotation was subsequently used to identify common themes among participants' viewpoints. The variables for analysis were the statement rankings produced by the clinicians. The purpose of the method was to identify orthogonal factors (i.e. at 90 degrees) representing different points of view among the clinicians. The clinician's loading on a factor indicated his/her shared viewpoint with other clinicians on the same factor. The clinicians loading on any one factor indicated a shared common viewpoint about their reasons for teaching by the similar rank ordering of their statement rankings. Positive scores indicated agreement, while negative scores indicated disagreement. Decreasing scores reflected less important views. Importantly, these factors were not considered as traits of the group, psychological or otherwise. They represented only common subjective opinions obtained at a single point in time in a cross-sectional survey method i.e. they reflected correlations between people not items. Q methodology yields ipsative rather then normative data in that a person reveals "individual" opinions rather than in comparison with another opinion [17]. Each identified factor was examined for distinguishing statements (those with scores that were significantly different at the p < 0.05 and p < 0.01 level from the same statement's score on other identified factors) [11].

## Results

### Demographic data

All 101 teachers were approached for participation. Of the 75 participants 18 (24%) were female and 57 (76%) were male with the following age distributions: 30–40 = 16 (21.3%), 41–55 = 46 (61.3%) and greater than 55 = 13 (17.3%). Most were staff specialists (n = 48, 64%) and 27 (36%) were visiting medical officers. Half of the participants were internists (n = 39, 52%), 12 (16%) were surgeons and 24 (32%) were other groups.

Of the 26 senior clinicians who did not participate 2 were women, 15 were visiting medical officers and 11 staff specialists, 16 were internists, 9 surgeons and 1 other. All but 2 of these clinicians fell in the 41–55 year age group. The participating teachers were seen to not differ significantly from the complete teaching list. Most nonparticipants were on leave during the study period.

### Factor analysis

The data sorted into four factors: Factor 1 (Table 2 accounting for 68 participants' sorts), Factor 2 (Table 3, 3 sorts), Factor 3 (Table 4, 2 sorts) and Factor 4 (Table 5, 2 sorts). In summary, Factor 1 the " I teach because..." factor, represented most participants agreeing with a range of positive statements about the value of teaching and disagreeing with statements phrased negatively about teaching. Highly ranked positive items included:

**Table 2:** Distinguishing statements for factor 1. (n = 68)

No.	Statement	Rank	Score
14	I teach because I want to help my students become good doctors	5	1.84*
3	I like the challenge of teaching students as effectively as possible	4	1.56*
57	I teach students to show them the correct way of clinical practice in my specialty	3	1.44*
1	I enjoy spending time with students in small groups	3	1.41*
7	I teach because I have been inspired to teach by my mentors	3	1.38*
54	I teach to ensure the students appreciate my specialty in a favourable way	2	1.04*
41	I teach to be challenged in my established views	2	1.02*
39	The teaching I had as a medical student has inspired me to want to teach	2	0.97*
32	I teach because I feel responsible for the student learning outcomes of my efforts	2	0.97*
52	I teach because I can enhance my knowledge and understanding of junior doctors	2	0.96*
58	I teach to ensure any false understanding of my specialty is not perpetuated	2	0.96*
40	I teach as a means of reviewing a topic area unfamiliar to me	1	0.90
55	I teach because it allows me to interact with students and show an appreciation of their position	1	0.77*
24	I teach because the course allows a deep approach to learning by the students	1	0.38
53	I teach because the interaction with students provides an opportunity for my opinions to be heard	1	0.31*
12	I teach because I enjoy the sense of performing in front of an audience	0	-0.22*
43	I teach because of the prestige it gives me with my peers	-1	-0.44
22	I don't teach because there is no recognition for what I do	-1	-0.78*
30	I don't teach because the setting in which I am expected to teach is inappropriate	-1	-0.86*
29	I don't teach because I believe the institution devalues teaching and learning	-2	-0.89
64	I don't teach as I don't approve of new teaching techniques	-2	-0.95
69	I don't teach as I feel my knowledge is out of date	-2	-1.07
62	I don't teach because one can't influence the behaviour of students for the better	-2	-1.10
19	I don't teach as my speciality is too 'cutting edge' to be relevant to students	-2	-0.11*
5	I don't feel any sense of duty to teach	-3	-1.12*
38	I don't teach because I am not concerned about the success of the clinical and/or medical school	-3	-1.15*
42	I don't teach because I find it unenjoyable	-3	-1.15*
63	I don't teach because teachers don't contribute to the formation of future doctors	-3	-1.15*
49	I don't teach because it fails to keep me up to date	-4	-1.19*
25	I don't teach as students make me feel inadequate	-4	-1.23*
45	I don't teach because interacting with students is boring	-4	-1.32*
4	I am bored by teaching	-5	-1.53*
66	I don't teach as it is a waste of time	-5	-1.78*

NB1. All P < 0.05 (* Indicates significance at P < 0.01)

**Table 3:** Distinguishing statements for factor 2 (n = 3)

No.	Statement	Rank	Score
21	I don't teach because there is no strong involvement of teaching staff in the design of the course	5	1.75*
2	I don't enjoy lecturing to large groups of students	4	1.49
66	I don't teach as it is a waste of time	1	0.50
7	I teach because I have been inspired to teach by my mentors	0	0.05
46	I teach because of the intellectual stimulation	-2	-0.71
10	I teach because I believe it is an appropriate service to my profession	-3	-1.33*

NB1. P < 0.05 (* Indicates significance at P < 0.01)

**Table 4:** Distinguishing statements for factor 3 (n = 2)

No.	Statement	Rank	Score
42	I don't teach because I find it unenjoyable	5	1.93
66	I don't teach as it is a waste of time	3	1.48
10	I teach because I believe it is an appropriate service to my profession	0	-0.23*
46	I teach because of the intellectual stimulation	-4	-1.70

NB1. P < 0.05 (* Indicates significance at P < 0.01)

**Table 5:** Distinguishing statements for factor 4 (n = 2)

No.	Statement	Rank	Score
36	My clinical load deters me from teaching	5	2.61*
23	Opportunities for academic promotion have nothing to do with my motivation	5	2.30
40	I teach as a means of reviewing a topic area unfamiliar to me	4	1.66*
9	I teach because it is a requirement of my employment contract	3	1.44
22	I don't teach because there is no recognition for what I do	1	0.18
29	I don't teach because I believe the institution devalues teaching and learning	0	-0.03*
64	I don't teach as I don't approve of new teaching techniques	0	-0.21
45	I don't teach because interacting with students is boring	0	-0.34*
69	I don't teach as I feel my knowledge is out of date	0	-0.37*
66	I don't teach as it is a waste of time	-1	-0.58*
56	I don't teach just because it is expected of me	-4	-1.54
18	I teach because there are opportunities for 'virtual' and/or 'online' and/or remote teaching	-5	-2.30

NB1. P < 0.05 (* Indicates significance at P < 0.01)

• helping students become good doctors

• enjoying the challenge of effective teaching

• valuing the presentation of one's own specialty

• enjoyment of small group teaching

• inspiration from mentors and past teachers

• liking to be challenged in one's views

• feeling responsible for students

• wanting to understand students.

The descending rank reflects less important views. These keen clinical teachers did not agree that they taught to "perform in front of an audience" and disagreed that teaching was boring, unsupported or a waste of time.

Factors 2, 3 and 4, the "I struggle to teach because ......" factor, represented 7 participant views in total, agreeing with a number of negative statements about teaching. Highly ranked negative items included:

• lack of involvement in course design

• lack of enjoyment in teaching

• clinical load deterring involvement in teaching.

It appears these 7 sorts are from those not motivated to teach. The software used prevented any further analysis of participants' demographic details and Q sorts.

## Discussion

This study shows that most senior medical clinicians, of diverse discipline, are motivated to teach medical students and that the main reason appears altruistic – a desire to help students become good doctors. A small, but not insignificant group, of senior clinicians do not want to teach, citing lack of involvement in the design in the course and excessive clinical load as negative motivators. Given the participants were taken from supposed active teaching lists this imbalance is not surprising.

Our results show that the majority of senior clinicians motivated to teach (factor 1) dwelt on a number of common items: inspiration from senior mentors, the altruistic role in development of junior doctors and the opportunity to highlight a specialty area. These items draw from concepts of goal directed or purposeful drivers of behaviour. Fulkerson highlighted some of these factors in a 1997 study [5]. These authors also found that the issue of rewards was important in motivating medical graduates to teach. Questions related to the value of rewards were included (such as payment, contracts and peer pressure) but these did not appear to be a prime motivator in our study group.

Factors 2 to 4 reflected small numbers of clinicians (seven in total) apparently disinterested in teaching. They cited issues such as disengagement with course material, lack of pleasure in teaching and excessive clinical load as impediments to teaching. While small in number, their views are worth examination as the dissenting opinion. These items are reinforcers (albeit negative reinforcers) of teaching behaviours. Schormair et al, 1992 [6] also drew attention to the lack of co-ordination of courses and the heavy burden of patient care and administrative tasks underlying the lack of motivation of some medical teachers to teach. In contrast to those who valued teaching, perhaps powerful mentors did not inspire this group during their own training.

The limitations of our study include the qualitative nature of the observations, the lack of breadth of sampling (focussing on a single hospital's clinical school affiliated staff and to some degree "reaching the converted"), the lack of inter-rater reliability assessments and the forced normalisation of the questions (perhaps unrestrained, participants may have skewed all statements to one pole or the other). Each member of the research team may have introduced the study differently (although there were common instructions). Although deidentified, the fact that each researcher recorded the views of their participants may have biased away from socially undesirable responses. The statements also cannot be considered exhaustive of all the possible reasons that may motivate a clinician to teach. A computerised questionnaire may also have been more efficient and interactive. It also must be remembered that motivation is an inherently complex construct and while Q methodology capture subjective views, it may not net all aspects of motivation.

There is also the issue of social desirability bias. Participants were aware that the evaluator was a colleague and would be aware of their results, perhaps skewing answers to those apparently more favourable. This may have contributed to an artificial multi-modal distribution. The questionnaire itself may also have contributed to an information bias. For example, in the heading it was requested to either strongly agree or strongly disagree rather than to give a more open level of agreement assessment. The questions asking why " I don't teach" to a group who are all allegedly teachers may lead to confusion, and again an observation bias or a classification bias. The incorrect placement of double negatives and even triple negatives may have led to noise, obscuring real differences and opinion.

### Implications

Clinical teachers are a valuable resource. They are essential to the successful teaching of medical students. Our study identified matters within the "ownership" of universities: engagement of clinical teachers in course design. This is an area ripe for action from medical schools and these data suggest that real inclusion would improve clinical teacher motivation. Rather more metaphysically, encouraging teachers to dwell on the inspirational models of their mentors may also enhance recruitment and retention. Opportunities to highlight special interests and to teach effectively would also be sensible strategies. Allowing clinical teachers to engage with small groups of students and to develop some understanding of student needs would meet the teachers' motivation to demonstrate their understanding of student experience. This sample suggests that contracts, money, a sense of duty and peer pressure play little part in motivating teachers. Previous research however, makes clear the important place of modest rewards such as Dean's teaching prizes. These data also underline the negative effect of service burden amongst clinical teachers. While this is not immediately under medical school control, universities can contribute meaningfully to discussions on balance of service and teaching commitments amongst health staff.

### Future research

This method could be replicated amongst more varied clinical teachers such as community practitioners and non-medical teachers to assess consistency of motivation. Further work is required to study the impact on clinical teacher workforce recruitment and retention through meeting these expressed motivations. We plan an intervention study to understand the effectiveness of tailoring teaching experience to these identified motivators. An understanding of intrinsic motivation is only helpful if it leads to higher teacher satisfaction, better quality teaching and retention in the workforce.

## Conclusion

Our inquiry suggests that promotion of teaching to senior clinicians is likely to have increased success if prospective teachers contribute to course development, sufficient time is allocated to teaching, memories of inspirational teachers are reawakened, the link between strong teaching and junior doctor outcomes is emphasised, and staff are reminded of the opportunity to 'advertise' their specialty. Medical schools face increasing difficulties staffing clinical programs and this study provides avenues to improve recruitment of senior medical staff to the teaching ranks.

## Competing interests

The author(s) declare that they have no competing interests.

## Authors' contributions

All authors contributed to design, data collection and paper preparation. DM and CO also conducted the data analysis.

## Pre-publication history

The pre-publication history for this paper can be accessed here:


